# Expression of long noncoding RNA uc.375 in bronchopulmonary dysplasia and its function in the proliferation and apoptosis of mouse alveolar epithelial cell line MLE 12

**DOI:** 10.3389/fphys.2022.971732

**Published:** 2022-08-30

**Authors:** Tianping Bao, Haiyan Zhu, Yafei Zheng, Jingjing Hu, Huifang Wang, Huaiping Cheng, Yuan Zhang, Zhaofang Tian

**Affiliations:** Department of Neonatology, The Affiliated Huaian No.1 People's Hospital of Nanjing Medical University, Huai'an, China

**Keywords:** long non-coding RNA uc.375, bronchopulmonary dysplasia, FoxA1, alveolar epithelial cell line, proliferation apoptosis

## Abstract

**Background:** According to our previous gene ChIP results, long noncoding RNA uc.375 was down-regulated in lung tissue of bronchopulmonary dysplasia (BPD) mice induced by hyperoxia. FoxA1 gene showed higher levels in lung tissue of BPD mice and is reported to promote the apoptosis of alveolar epithelial cells. We aimed to clarify the expression pattern of uc.375 in BPD and explore the interaction between uc.375 and FoxA1.

**Methods:** Newborn mice were placed in a 95% high-oxygen environment for 7 days. Lung tissue samples from mice were used for lncRNA microarray to screen BPD related lncRNAs. Mouse alveolar epithelial cell line MLE 12 was stably transfected with uc.375 and FoxA1 silencing or overexpression lentiviral vectors. The proliferation activity of MLE 12 cells was detected by a cell counting kit 8 (CCK-8) assay. MLE 12 cell apoptosis was determined by Hoechst/PI staining and flow cytometry analysis. The protein levels of Cleaved Caspase-3, FoxA1, SP-C and UCP2 were investigated by western blot. The relative mRNA expression levels were detected by quantitative real-time PCR.

**Results:** uc.375 is mainly distributed in the nucleus of alveolar epithelial cells, as revealed by *In Situ* Hybridization assay results. uc.375 was lowly expressed in the lung tissues of BPD mice. According to the results of CCK-8 assay, analysis of Hoechst/PI staining and western blotting, uc.375 silencing inhibited cell proliferation, facilitated apoptosis of MLE 12 cells, promoted caspase 3 and FoxA1 expression, and inhibited the expression of SP-C and UCP2. On the contrary, after overexpressing uc.375, the opposite results were obtained. Silencing FoxA1 inhibited MLE 12 apoptosis, promoted proliferation, inhibited apoptosis-related factor caspase 3, and promoted the expression of SP-C and UCP2. FoxA1 silencing also reversed the effect induced by uc.375 knockdown on the proliferation and apoptosis of MLE 12 cells.

**Conclusion:** Based on the biomedical images-derived analysis results, uc.375 negatively regulates FoxA1 expression, affects alveolar development, and plays an important role in the initiation and progression of BPD, providing a new molecular target for the prevention and treatment of BPD.

## Highlights

LncRNA uc.375 is downregulated in BPD
uc.375 silence inhibits MLE 12 cell proliferation and facilitates apoptosis
uc.375 negatively regulates FoxA1 expression
Silencing FoxA1 inhibits MLE 12 apoptosis, promotes proliferation

## Introduction

Bronchopulmonary dysplasia (BPD) is a common chronic lung disease in preterm infants, and its incidence is as high as 52% in extremely low birth weight infants (Natarajan et al., 2012; Villosis et al., 2021). With the increasing maturity of critical care and management techniques for premature infants, the survival rate of very low and extremely low birth weight infants has increased significantly, but with it, the incidence of BPD has also shown a continuous upward trend (Jensen and Schmidt, 2014). Children with this disease not only suffer from repeated respiratory infections and varying degrees of pulmonary dysfunction, but also face increased risk of asthma and chronic obstructive pulmonary disease (COPD) in adulthood (Teune et al., 2012). Therefore, BPD has become an important medical problem that endangers the health of newborns, affects the quality of our nationals, and causes a heavy family and socioeconomic burden.

LncRNA is a type of non-coding transcript longer than 200 nucleotides and without the ability to encode proteins. In recent years, with the rapid development of genetic information technology and the wide application of next-generation sequencing and gene chip technology, the understanding of the pathogenesis of many human diseases has gradually expanded to the level of lncRNA, which has become a research hotspot in many fields of life medicine. It has also become a new direction for us to explore the pathogenesis of BPD. LncRNAs have abundant biological functions in cell development and metabolism, such as genetic imprinting (Kretz et al., 2013), chromatin modification (Tsai et al., 2010), cell cycle regulation (Hung et al., 2011), transcription regulation (Huarte et al., 2010), and participation in mRNA degradation (Gong and Maquat, 2011). These are closely related to the occurrence, development and prevention of many human diseases. Studies on the role and mechanism of lncRNA in lung development and lung diseases have become a hot spot of increasing attention by scholars. Herriges et al. (2014) identified 363 lncRNAs in embryonic mouse lungs, which interact with transcription factors spatially to regulate lung development. The lack of certain lncRNAs in the lungs can lead to alveolar capillary dysplasia and pulmonary vein dislocation (ACD/MPV) (Szafranski et al., 2013). Studies have also confirmed that lncRNA plays the role of “inducers and terminators” of vascular development (Viereck et al., 2015), affects the biological functions of endothelial cells, and can be used as a target for vascular diseases (Fiedler et al., 2015). Although there have been preliminary studies of lncRNA in the field of lung development, further exploration of the role and mechanism of lncRNA in the formation of BPD will inevitably promote the in-depth understanding of the pathogenesis of BPD.

In this study, we aimed to explore the function and molecular mechanism of the lncRNA uc.375 in BPD based on lncRNA microarray, bioinformatics, and functional studies. We hypothesized that lncRNA uc.375 promoted MLE 12 cell growth *in vitro*. In summary, this study clarified the exact mechanism of uc.375 in the occurrence and development of BPD. We identified that it may provide a new molecular target for the prevention and treatment of BPD.

## Methods

### RT-PCR

Total RNA was isolated from tissues and cells using TRIzol Reagent (Applygen, Beijing, China) in accordance with the manufacturer’s instructions. qRT-PCR was carried by using Reverse Transcription Kit (Haigene, Harbin, China). After reverse transcription, quantitative real-time PCR analysis was performed as previously described (Zhao et al., 2017).

### Cell culture and transfection

MLE 12 cell line was obtained from Bei Na Chuang Lian Biotechnology Research Institute (Beijing, China) and followed their instructions to culture at 37°C. Stably uc.375 or FoxA1 silencing cell lines were screened out as previously reported (Liu et al., 2018). The cells were transfected with 10 pmol/ml of uc.375 or FoxA1 short-hairpin RNA, or negative control (Sigma-Aldrich) and selected for 4 weeks with neomycin (1,000 μg/ml). To up-regulate uc.375 or FoxA1, the MLE 12 cells were transfected with uc.375 or FoxA1 overexpression lentiviral vector according to the manufacturer’s protocols.

### CCK-8 assay

Cell viability was detected by CCK-8 kit (MedChemExpress, Shanghai, China). Cells (3,000/well) were cultured in 96-well plates for 48 h. After incubated with 10 μl CCK8 for 3–4 h, the absorbance was measured at 450 nm using GloMax^®^ System (Promega, WI, United States) (Chen et al., 2020).

### Hoechst PI staining

The single cell suspension was prepared routinely. The final concentration of Hoechst 33,258 was 1 mg/ml, and the final concentration of Hoechst 33,258 was dissolved in water at 37°C for 7 min. The dye solution was cooled on ice, and centrifuged and overhung. The final concentration of PI solution was 5 mg/ml, and the ice bath was added. The dye solution was centrifuged and washed once by PBS, and the Blue fluorescence at 400–500 nm and the red fluorescence at 630 nm or above were recorded by direct external laser Flow cytometry analysis.

### Western blot

Total proteins were isolated from cells using a total protein extraction kit (Keygen, Nanjing, China). A total of 40 µg protein was separated using SDS-PAGE and transferred onto polyvinylidene difluoride (PVDF) membranes and then blocked with 5% fat-free milk at room temperature for 2 h. The immune-blot was incubated with primary antibody (1:1,000 dilution; Cell signaling) including anti-caspase 3 (ab184787, 1:2000, Abcam), anti-FoxA1 (ab170933, 1:1,000, Abcam), anti-SP-C (PA5-119603, 1:1,000, Thermo Fisher), anti-UCP2 (PA5-77553, 1:1,000, Thermo Fisher) and GAPDH (1:1,000 dilution; Cell signaling) was used as a control. The signals were detected using a Super ECL Plus Kit (Keygen) and determined by quantitative analysis using UVP software (UVP, LLC, Upland, CA, United States).

### Flow cytometry assay

The apoptosis rate of MLE 12 cells was detected by Annexin V-FITC/PI Apoptosis Detection Kit (Yeasen, Shanghai, China) via flow cytometry. After cultured for 48 h, cells were collected, following by staining with 10 μL of Annexin V-FITC and PI. Then cells were analyzed by flow cytometer (BD, New Jersey, United States) and quantified by the FlowJo software (Tree Star, United States) (Wang et al., 2020).

### *In Situ* Hybridization (ISH)

ISH assay was performed to detect the localization of uc.375 in MLE 12 cells. The cell slides were fixed with 4% paraformaldehyde for 20 min and added with 20 μg/ml proteinase K for digestion. The hybridization was conducted using uc.375 probes provided by the Boster Biological Technology (Wuhan, China). Subsequently, the slides were cultured with anti-DIG reagents at 37°C overnight. The images were photographed with a Zeiss LSM510 microscope.

### Statistical analysis

Data are shown as the mean ± SD. SPSS 21.0 (IBM Corp., NY, United States) was used for statistical analysis of all data. *t*-test was used for comparison between two groups, and one-way ANOVA and Tukey’s post-tests were used for comparison among multiple groups. The level of significance was set at *p* < 0.05.

## Results

### Analysis of uc.375 expression characteristics

**FIGURE 1:**
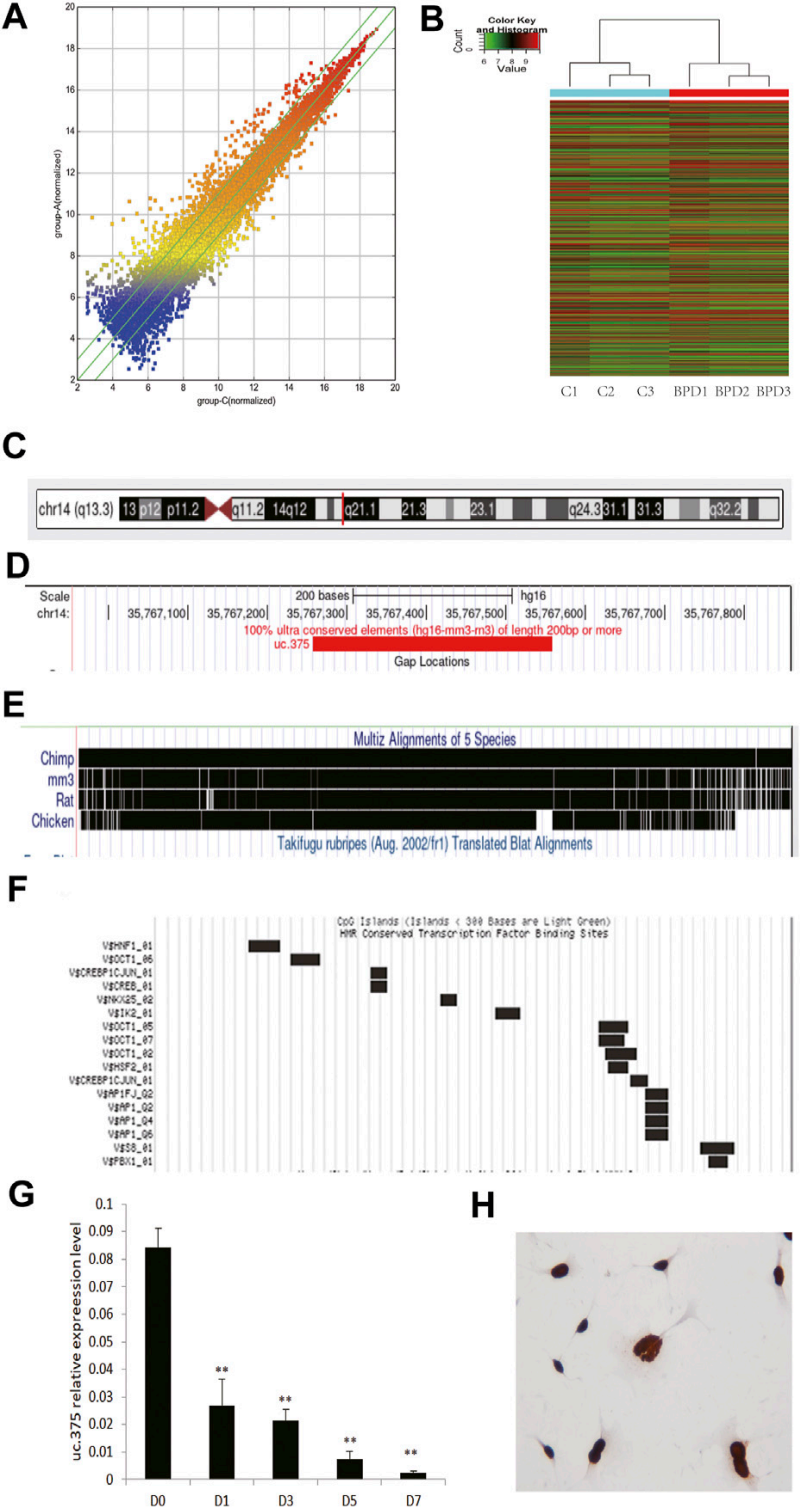
Analysis of uc.375 expression characteristics. **(A)** Scatter analysis chart of lncRNAs expression in hyperoxia-induced BPD lung tissue and normal lung tissue. **(B)** Cluster analysis. **(C-F)** uc.375 Bioinformatics characteristics. **(G)** The relative expression level of uc.375 was detected by qRT-PCR in lung tissues at different time points (D0, D1, D3, D5, D7) during BPD modeling. **(H)** The localization of uc.375 in MLE 12 cells. **p < 0.01.

We placed newborn mice in a 95% high-oxygen environment for 7 days, and the pathological structure of the mouse lung successfully simulated the characteristics of human BPD. We selected lung tissue samples of the BPD group and the air control group as the test objects, and used lncRNA microarray technology (Mouse lncRNA Microarray V3.0, Arraystar, probes covering 35,923 long non-coding RNAs and 24,881 coding genes) to screen BPD related lncRNAs. The results suggest that the expression of lncRNA in lung tissue of BPD induced by hyperoxia is quite different from that in normal lung tissue (Figure 1A: Scattered analysis diagram; Figure 1B: Cluster analysis), suggesting that these differences in lncRNA may be the molecular basis for the development of BPD. When analyzing the differentially expressed lncRNAs, we found that there were 140 upregulated lncRNAs with five times or more, and 71 downregulated lncRNAs with 5 times or more.

We also conducted a bioinformatics analysis on uc.375 and identified that uc.375 has a full length of 300bp and is located on human chromosome 14q13 (Figure 1C). It is well-conserved among mammals (Figures 1D,E). There are many transcriptional factor binding sites in the chromosome region where the uc.375 sequence is located (Figure 1F), suggesting that it may have a potentially powerful transcriptional regulatory effect. We further investigated the expression of uc.375 at different time points in the development of BPD. We exposed newborn mice to 95% oxygen, and qRT-PCR was used to detect the expression of uc.375 in lung tissue at different time points (D0, D1, D3, D5, D7 day). The results showed that uc.375 expression decreased gradually with time (Figure 1G). We detected the localization of uc.375 in MLE 12 cells by RNA ISH, and we found that uc.375 existed in the nucleus (Figure 1H).

### The effect of uc.375 silencing on MLE 12 cells function and FoxA1 expression

**FIGURE 2:**
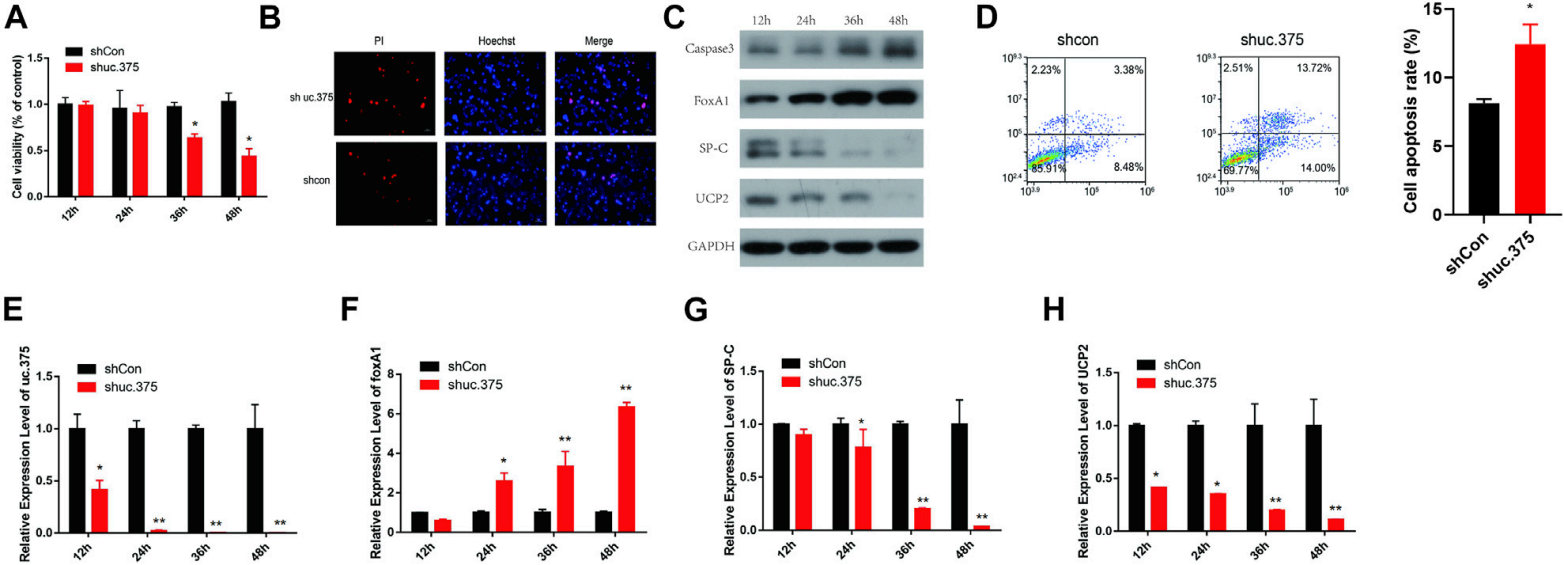
The effect of uc.375 silencing on MLE 12 cells function and FoxA1 expression. **(A)** The proliferation activity of MLE 12 cells was detected by CCK-8 assay. **(B)** The cell apoptosis was determined by Hoechst/PI staining in MLE 12 cells. **(C)** The protein levels of caspase3, FoxA1, SP-C and UCP2 were investigated by western blot in MLE 12 cells. **(D)** The cell apoptosis was determined using low cytometry assay in MLE 12 cells. E-H. The relative mRNA expression levels of uc.375, FoxA1, SP-C and UCP2 were detected by qRT-PCR in MLE 12 cells. *p < 0.05, **p < 0.01 vs. shCon.

To assess the effect of uc.375 silencing on MLE 12 cells function, MLE 12 cells were stably transfected with uc.375 silent lentiviral vector to knock down uc.375. CCK-8 assay was conducted and cell viability was diminished by sh-uc.375 (Figure 2A). In addition, the effect of sh-uc.375 on cell apoptosis was assessed by Hoechst/PI staining and flow cytometry. The cell apoptosis was remarkably promoted by sh-uc.375 (Figures 2B,D). Moreover, the protein levels of caspase 3, FoxA1, SP-C and UCP2 were investigated by western blot in MLE 12 cells. The results showed that SP-C and UCP2 were suppressed, whereas caspase 3 and FoxA1 were markedly stimulated by sh-uc.375 silencing (Figure 2C). In addition, the qRT-PCR results confirmed that uc.375 level was significantly repressed by sh-uc.375, the SP-C and UCP2 mRNA expression were suppressed, whereas FoxA1 was markedly stimulated by sh-uc.375 (Figures 2E–H). Taken together, uc.375 silencing inhibited cell proliferation and promoted apoptosis.

### The effect of uc.375 overexpression on the function of MLE 12 cells

**FIGURE 3:**
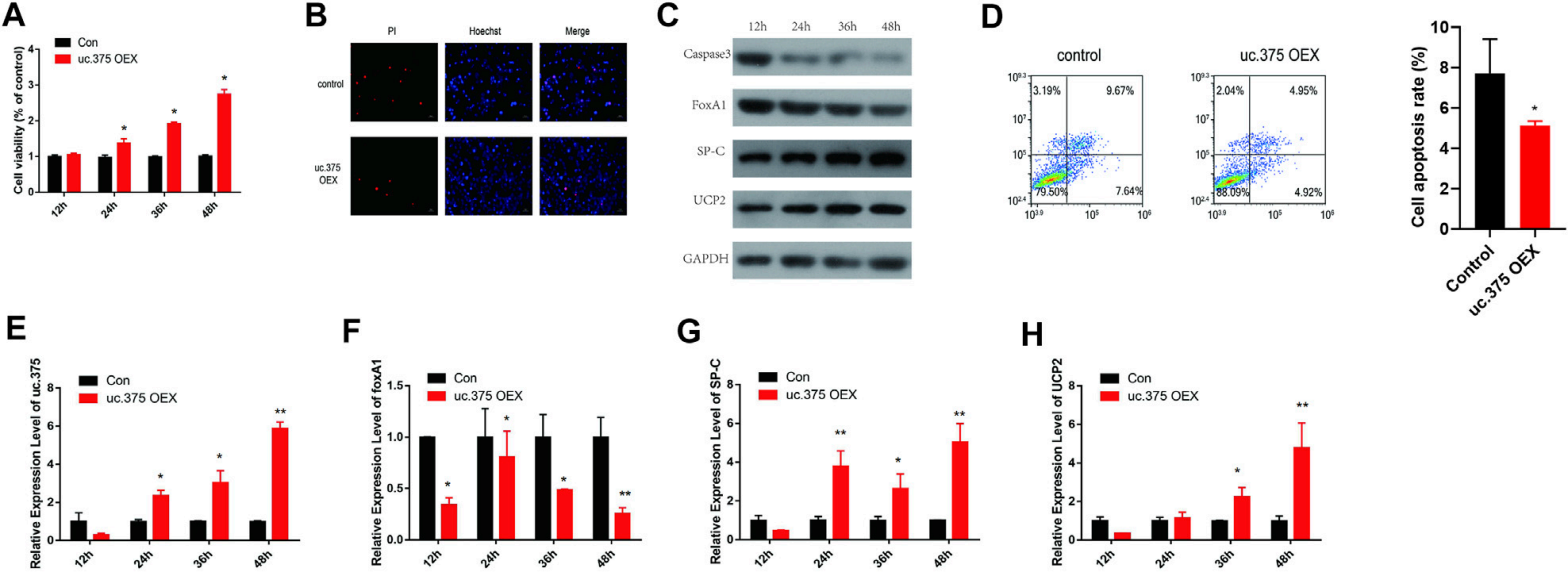
The effect of uc.375 overexpression on the function of MLE 12 cells. **(A)** The proliferation activity of MLE 12 cells was detected by CCK-8 assay. **(B)** The cell apoptosis was determined by Hoechst/PI staining in MLE 12 cells. **(C)** The protein levels of caspase3, FoxA1, SP-C and UCP2 were investigated by western blot in MLE 12 cells. **(D)** The cell apoptosis was determined using low cytometry assay in MLE 12 cells. **(E-H)** The relative mRNA expression levels of uc.375, FoxA1, SP-C and UCP2 were detected by qRT-PCR in MLE 12 cells. *p < 0.05, **p < 0.01 vs. Con.

To further assess the effect of uc.375 overexpression on MLE 12 cell functions, MLE 12 cells were stably transfected with uc.375 overexpression lentiviral vector to overexpress uc.375. CCK-8 assay was conducted and cell viability was increased by uc.375 overexpression (Figure 3A). In addition, the effect of uc.375 overexpression on cell apoptosis was assessed by Hoechst/PI staining and flow cytometry. The cell apoptosis was remarkably reduced by uc.375 overexpression (Figures 3B,D). Moreover, the protein levels of caspase 3, FoxA1, SP-C and UCP2 were investigated by western blot in MLE 12 cells. The results showed that SP-C and UCP2 were increased, whereas caspase 3 and FoxA1 were markedly inhibited by uc.375 overexpression (Figure 3C). In addition, the qRT-PCR results confirmed that uc.375 level was significantly elevated by uc.375 overexpression, the SP-C and UCP2 mRNA expression were stimulated, whereas FoxA1 mRNA expression was markedly suppressed by uc.375 overexpression (Figures 3E–H). Overall, uc.375 overexpression promoted cell proliferation and repressed apoptosis.

### Effects of uc.375 silencing and FoxA1 silencing on the function of MLE 12 cells

**FIGURE 4:**
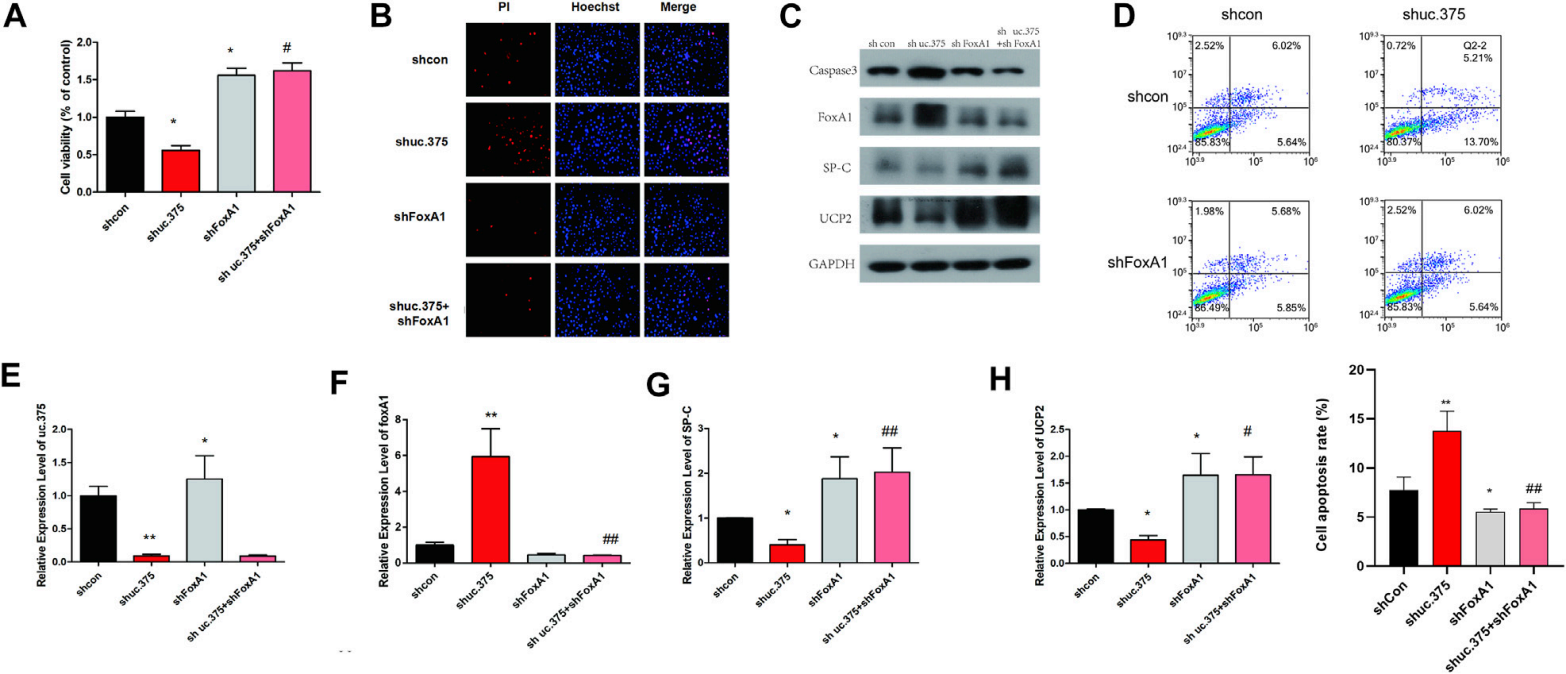
Effects of uc.375 silencing and FoxA1 silencing on the function of MLE 12 cells. **(A)** The proliferation activity of MLE 12 cells was detected by CCK-8 assay. **(B)** The cell apoptosis was determined by Hoechst/PI staining in MLE 12 cells. **(C)** The protein levels of caspase3, FoxA1, SP-C and UCP2 were investigated by western blot in MLE 12 cells. **(D)** The cell apoptosis was determined using low cytometry assay in MLE 12 cells. **(E-H)** The relative mRNA expression levels of uc.375, FoxA1, SP-C and UCP2 were detected by qRT-PCR in MLE 12 cells. *p < 0.05, **p < 0.01 vs sh-Con. #*p* < 0.05, ##*p* < 0.01 vs.. sh uc.375.

To further evaluate the effect of uc.375 silencing and FoxA1 silencing on MLE 12 cells function, MLE 12 cells were stably co-transfected with uc.375 silencing/FoxA1 silencing to knock down uc.375 and FoxA1. CCK-8 assay was conducted and cell viability was increased by FoxA1 silencing compared with the control group. The viability of MLE 12 cells was increased in the shuc.375 + shFoxA1 group compared with the shuc.375 group (Figure 4A). In addition, the cell apoptosis was assessed by Hoechst/PI staining and flow cytometry. The cell apoptosis was notably reduced by FoxA1 silencing compared with the control group. FoxA1 silencing also reversed the enhanced effect of uc.375 silencing on cell apoptosis (Figures 4B,D). Moreover, the protein levels of caspase 3, FoxA1, SP-C and UCP2 were detected by western blot in MLE 12 cells. The results showed that SP-C and UCP2 were increased, whereas caspase 3 and FoxA1 were markedly inhibited by FoxA1 silencing compared with the control. The uc.375 silencing induced elevation in Caspase 3 and decrease in SP-C and UCP2 levels were all reversed after FoxA1 silencing (Figure 4C). In addition, the qRT-PCR results confirmed that uc.375 level was significantly reduced by uc.375 silencing, and was not significantly affect by FoxA1 silencing. The SP-C and UCP2 mRNA expression were stimulated by FoxA1 silencing, and FoxA1 silencing was revealed to reverse the inhibitory effect of uc.375 silencing on SP-C and UCP2 mRNA expression (Figures 4E–H). Taken together, FoxA1 silencing stimulated cell proliferation and suppressed apoptosis.

### Effects of uc.375 overexpression and FoxA1 overexpression on the function of MLE 12 cells

**FIGURE 5:**
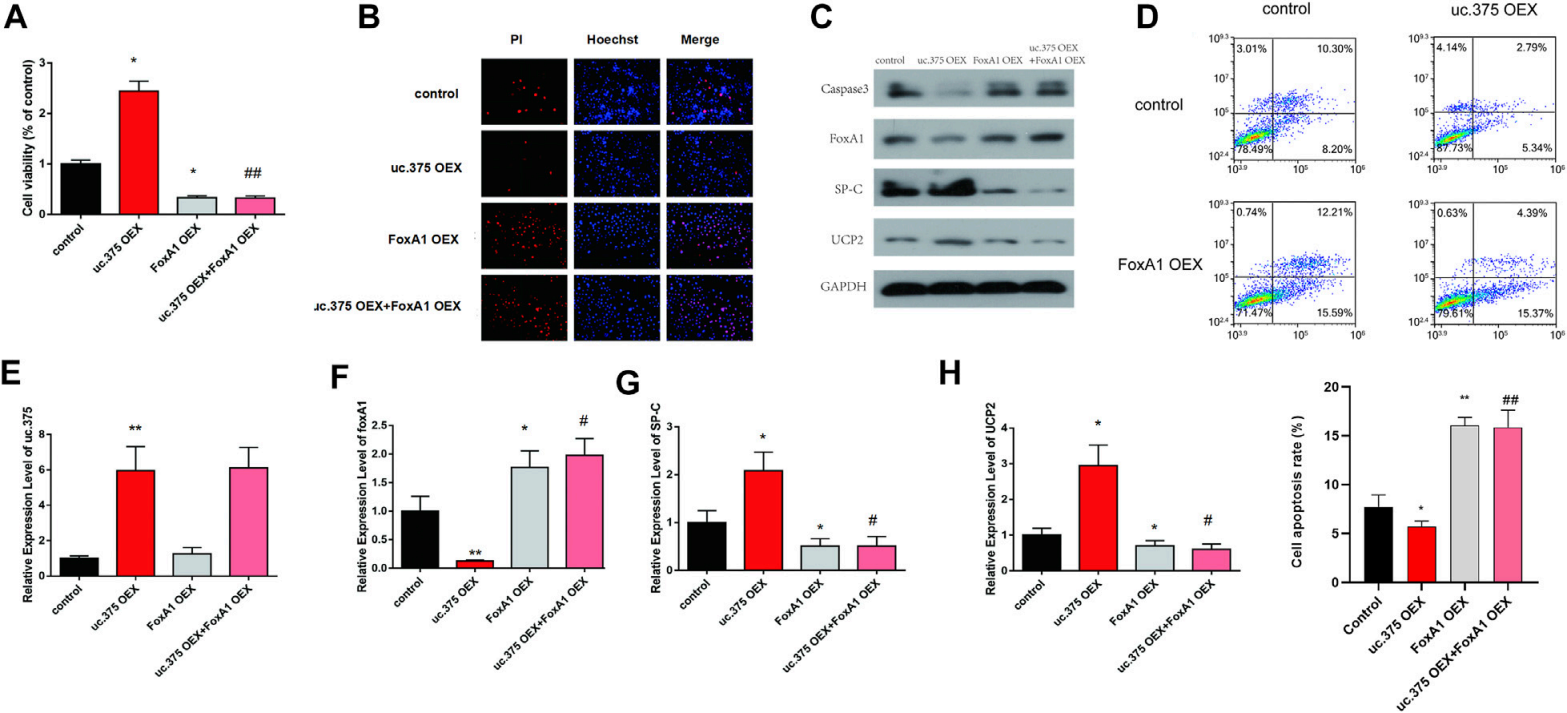
Effects of uc.375 overexpression and FoxA1 overexpression on the function of MLE 12 cells. **(A)** The proliferation activity of MLE 12 cells was detected by CCK-8 assay. **(B)** The cell apoptosis was determined by Hoechst/PI staining in MLE 12 cells. **(C)** The protein levels of caspase3, FoxA1, SP-C and UCP2 were investigated by western blot in MLE 12 cells. **(D)** The cell apoptosis was determined using low cytometry assay in MLE 12 cells. **(E-H)** The relative mRNA expression levels of uc.375, FoxA1, SP-C and UCP2 were detected by qRT-PCR in MLE 12 cells. *p < 0.05, **p < 0.01 vs control. #*p* < 0.05, ##*p* < 0.01 vs uc.375 OEX.

To further evaluate the effect of uc.375 overexpression and FoxA1 overexpression on MLE 12 cells function, MLE 12 cells were stably co-transfected with uc.375 overexpression/FoxA1 overexpression to overexpress uc.375 and FoxA1. CCK-8 assay was conducted and cell viability was decreased by FoxA1 overexpression compared with the control group. The viability of MLE 12 cells was also decreased in the uc.375 OEX + FoxA1 OEX group compared with the uc.375 OEX group (Figure 5A). In addition, the cell apoptosis was assessed by Hoechst/PI staining and flow cytometry. The cell apoptosis was notably increased by FoxA1 overexpression compared with the control. The apoptosis rate of MLE 12 cells was also significantly elevated in the uc.375 OEX + FoxA1 OEX group compared with the uc.375 OEX group (Figures 5B,D). Moreover, the protein levels of caspase 3, FoxA1, SP-C and UCP2 were detected by western blot in MLE 12 cells. The results showed that SP-C and UCP2 were reduced, whereas caspase 3 and FoxA1 were markedly increased by FoxA1 overexpression compared with the control. FoxA1 overexpression was also demonstrated to reverse the enhancement of uc.375 overexpression on SP-C and UCP2 protein levels and the inhibition of uc.375 overexpression on caspase 3 protein expression (Figure 5C). In addition, the qRT-PCR results confirmed that uc.375 level was significantly elevated by uc.375 overexpression and showed no significant change after FoxA1 overexpression. The SP-C and UCP2 mRNA expression were inhibited by FoxA1 overexpression, and their expression was reduced in the uc.375 OEX + FoxA1 OEX group compared with the uc.375 OEX group (Figures 5E–H). Taken together, FoxA1 overexpression suppressed cell proliferation and stimulated apoptosis.

## Discussion

At present, the pathogenesis of BPD has not yet been fully elucidated. Most scholars believe that BPD is a multifactorial disease-causing syndrome. Alveolar epithelial cell apoptosis dominates immature lung injury, and the pathological feature of BPD is arrest of alveolar developmental with increased alveolar epithelial cell apoptosis (Zhang et al., 2020). Alveolar epithelial cell II is a stem cell of alveolar epithelial cells, which has the ability of immortal proliferation and differentiation (Fehrenbach, 2001; Toulmin et al., 2021). During lung development, alveolar epithelial cell II continuously differentiates into alveolar epithelial cell I (AEC I) to promote alveolar formation and complete alveolar remodeling after epithelial injury (Wang et al., 2013). Alveolar epithelial cell II is an important effector cell in the pathological process of lung injury. Its function and status determine the pathological outcome of lung injury. The survival and apoptosis of alveolar epithelial cells affect the outcome of lung injury repair.

Lung development is an intricate process. From a genetic perspective, it is the result of the expression, regulation and interaction of key lung development genes at different times and in different spaces. A slight abnormality in any link may disrupt normal lung development and cause varying degrees lung injury (Ameis et al., 2017). With the rapid development of molecular genetic research, a number of genes closely related to BPD have been discovered, such as VEGF-α, TGF-β, IGF-1, fibronectin 1, p21, FoxA1, etc. To a certain extent, the changes of the expression levels of these genes in BPD lung tissues can reflect the development trend of BPD (Ameis et al., 2017). Studies have reported that non-coding RNA is also involved in the pathogenesis of BPD. For example, miRNA-29 is highly expressed in the lung tissue of newborn mice with BPD, and it participates in the occurrence and development of BPD by down-regulating the expression of Ntrk2 and disrupting various biological processes of lung development (Dong et al., 2012). Recent studies have also reported that lncRNA MALAT1 can protect BPD by inhibiting cell apoptosis (Cai et al., 2017). Although scholars at home and abroad have done a lot of meaningful work on the pathogenesis of BPD, providing molecular basis and clues for understanding the formation process of BPD, the pathogenesis of BPD is still not fully elucidated, and the existing pathogenesis cannot provide effective clinical interventions, thus in-depth exploration of new mechanisms of BPD pathogenesis is urgent.

uc.375 is a lncRNA with unknown function that is highly conserved among different species, differentially lowly expressed in BPD lung tissue, mainly localized in alveolar type II epithelial cells, and obtained through microarray technology screening. Regarding the biological information, function and role of uc.375 in BPD, there are currently no relevant reports at home and abroad, and there is no report on the function and mechanism of uc.375 involved in the process of BPD alveolar development. Our research found that the expression of uc.375 in lung tissue of BPD mice was gradually decreased with time. Silencing uc.375 in the mouse alveolar type II epithelial cell line (MLE 12) can significantly promote its apoptosis and inhibit its proliferation, while uc.375 overexpression showed opposite effects. The expression of FoxA1 was negatively regulated by uc.375, and FoxA1 overexpression or silencing showed no significant impact on uc.375 expression. uc.375 affects the development of alveoli, and plays an important role in the development of BPD by regulating FoxA1.

The FoxA family is a class of proteins with important functions, which are involved in the regulation of cell proliferation, differentiation, embryonic development and other important life activities. Some members of the FoxA family play an important role in the regulation of apoptosis-related genes. For example, FoxA2 can significantly inhibit the expression of SP-A in A549, and SP-A is an apoptosis inhibitor of alveolar epithelial cells (White and Strayer, 2002). 2-Acetylaminofluorene promotes bile duct cell apoptosis through FoxA3 (Bisgaard et al., 1996). FoxA1’s nuclear localization sequence, DNA binding domain, and NH2 terminal transcription activation are highly homologous to FoxA2 and 3. FoxA1 is closely related to the embryonic development of endoderm-derived tissues and organs. Lee et al. (2005) reveal that the initiation of liver development during embryonic development is FoxA1-dependent, and the development of fetal lungs is also regulated by FoxA1, and FoxA1 is only found in the lungs. It is expressed in airway epithelial cells and alveolar type II epithelial cells in tissues (Besnard et al., 2004). Studies have also found that the loss of FoxA1 in mouse lung tissue alveolar epithelial cells and bronchial Clara cells will delay cell development and maturation. In addition, the expression of SP-B and SP-C in mice lacking FoxA1 is significantly lower than that in wild-type mice, eventually leading to increased susceptibility to respiratory diseases after birth (Besnard et al., 2005; Paranjapye et al., 2020). It is more reported in the literature that FoxA1 inhibits its expression by combining with the anti-apoptotic genes bcl2 and FoxA1 binding elements in the promoter region of UCP2, thereby promoting alveolar epithelial cells apoptosis and playing a role in BPD (Xu et al., 2014). In this study, we identified that FoxA1 is the regulatory target of uc.375. The expression of FoxA1 was increased in lung tissue of BPD mice with time after silencing uc.375, and FoxA1 silencing promoted the proliferation and suppressed apoptosis of MLE 12 cells, while its overexpression showed opposite effects. Moreover, we also found that FoxA1 silencing reversed the effect of uc.375 knockdown on MLE 12 cell proliferation and apoptosis. Similarly, FoxA1 overexpression also rescued the effect exerted by uc.375 upregulation on MLE 12 cells.

## Conclusion

In summary, our study used biomedical images such as Hoechst PI staining and ISH, elucidating that uc.375 negatively regulates FoxA1 expression, affects the development of alveoli, and plays an important role in the occurrence and development of BPD. It will provide a novel idea and potential target for further study on prevention and treatment of BPD.

## Data Availability

The original contributions presented in the study are included in the article/Supplementary Material, and further inquiries can be directed to the corresponding author.
