# Supplementary material for: Expression of long noncoding RNA uc.375 in bronchopulmonary dysplasia and its function in the proliferation and apoptosis of mouse alveolar epithelial cell line MLE 12
Source: Front Physiol. 2022 Aug 30;13:971732. doi: 10.3389/fphys.2022.971732 (PMC9468891; doi:10.3389/fphys.2022.971732)
Supplement: Supplementary file 1 [file Table1.DOCX]

Raw data were shared at https://www.jianguoyun.com/p/DZJVGiUQ-tvKChj97b8EIAA.
